# Cost and impact of scaling up interventions to save lives of mothers and children: taking South Africa closer to MDGs 4 and 5

**DOI:** 10.3402/gha.v8.27265

**Published:** 2015-04-21

**Authors:** Lumbwe Chola, Yogan Pillay, Peter Barron, Aviva Tugendhaft, Kate Kerber, Karen Hofman

**Affiliations:** 1 PRICELESS – MRC/Wits Rural Public Health and Health Transitions Research Unit (Agincourt), School of Public Health, Faculty of Health Sciences, University of the Witwatersrand, Johannesburg, South Africa; 2 South Africa National Department of Health, Pretoria, South Africa; 3 School of Public Health, Faculty of Health Sciences, University of the Witwatersrand, Johannesburg, South Africa; 4 Save the Children, Cape Town, South Africa; 5 School of Public Health, University of the Western Cape, Bellville, South Africa

**Keywords:** child health, maternal health, cost and cost analysis

## Abstract

**Background:**

South Africa has made substantial progress on child and maternal mortality, yet many avoidable deaths of mothers and children still occur. This analysis identifies priority interventions to be scaled up nationally and projects the potential maternal and child lives saved.

**Design:**

We modelled the impact of maternal, newborn and child interventions using the Lives Saved Tools Projections to 2015 and used realistic coverage increases based on expert opinion considering recent policy change, financial and resource inputs, and observed coverage change. A scenario analysis was undertaken to test the impact of increasing intervention coverage to 95%.

**Results:**

By 2015, with realistic coverage, the maternal mortality ratio (MMR) can reduce to 153 deaths per 100,000 and child mortality to 34 deaths per 1,000 live births. Fifteen interventions, including labour and delivery management, early HIV treatment in pregnancy, prevention of mother-to-child transmission and handwashing with soap, will save an additional 9,000 newborns and children and 1,000 mothers annually. An additional US$370 million (US$7 per capita) will be required annually to scale up these interventions. When intervention coverage is increased to 95%, breastfeeding promotion becomes the top intervention, the MMR reduces to 116 and the child mortality ratio to 23.

**Conclusions:**

The 15 interventions identified were adopted by the National Department of Health, and the Health Minister launched a campaign to encourage Provincial Health Departments to scale up coverage. It is hoped that by focusing on implementing these 15 interventions at high quality, South Africa will reach Millennium Development Goal (MDG) 4 soon after 2015 and MDG 5 several years later. Focus on HIV and TB during early antenatal care is essential. Strategic gains could be realised by targeting vulnerable populations and districts with the worst health outcomes. The analysis demonstrates the usefulness of priority setting tools and the potential for evidence-based decision making in the health sector.

In 2013, with 2 years left to the Millennium Development Goals (MDG) deadline, the South African National Department of Health (NDOH) commissioned a National Countdown to 2015, an initiative aimed at strengthening efforts to reach MDG 4 (reduction in child mortality) and MDG 5 (reduction in maternal mortality), and also to generate traction for the post-2015 maternal and child health (MCH) agenda. Undertaken as part of this initiative was an exercise to identify key interventions that would be most effective in saving additional lives of mothers, newborns and children, particularly in the 2 years leading up to the MDG deadline. In this paper, we provide details of the process undertaken to identify these priority interventions and the additional maternal, newborn and child lives that could be saved if these interventions were scaled up.

The study was commissioned based on the realisation that South Africa would not achieve MDGs 4 and 5 at the current trajectory, despite significant progress made since 2005 to reduce maternal and child mortality (1, 2). This progress has been achieved largely through health system strengthening interventions since 1994, as well as a significant response to the HIV epidemic, especially since 2009 (3). Healthcare has been free to all pregnant women and children since 1996, and 90% of births occur in health facilities (4). More recently, the focus on primary healthcare re-engineering has been designed to improve access to and quality of healthcare (5). Routine pneumococcal and rotavirus vaccines have been in policy since 2009 and World Health Organisation (WHO) guidelines on exclusive breastfeeding and appropriate complementary feeding were adopted in 2011 (6). In addition, child support grants targeting vulnerable children have had a positive impact on health (7).

However, many challenges still remain. Immunisation coverage at national level masks inequities across the 52 districts, with some districts recording suboptimal coverage (8). Over 40% of maternal deaths are HIV related (9). Antenatal care (ANC) attendance before 20 weeks is about 50% nationally (10). Teenage pregnancy is high; almost 8% of all births (11) and an estimated 50% of all abortions are performed illegally (12). Maternal and child mortality remains higher than other middle-income settings. Compared to Brazil, Russia, India and China (BRIC countries), South Africa has the highest under-five mortality rate (U5MR), and achieved the lowest reduction in child mortality (20%) between 1990 and 2011, compared to India (69%) and Brazil (72%) (13). There is, therefore, need for equitable and high-quality implementation of key interventions to reduce avoidable deaths in South Africa. Studies have shown the potential impact and resource requirements of scaling up priority MCH interventions globally (14–19).

This analysis aimed to identify priority interventions that can be scaled up between 2014 and 2015, project the additional number of lives that can potentially be saved, and estimate the resource requirements necessary to scale up the priority interventions. The study was initiated through a process of engagement between policymakers in the NDOH, researchers from the University of the Witwatersrand, the WHO in South Africa and other stakeholders. A decision was made to use the Lives Saved Tool (LiST) to assist in priority setting, given that the tool has been widely used for this purpose (14–19). Consultation with stakeholders was maintained throughout the study, with methodological support provided by the developers of the tool (The Futures Group – http://futuresgroup.com/).

## Methods

Firstly, an assessment was undertaken to examine the progress that South Africa had made with regard to reducing maternal, newborn and child mortality in the last decade. This was done through a review of the coverage of known essential interventions and the impact that they had had on maternal and child mortality in the country. Secondly, information was gathered on the causes of deaths and trends in mortality indicators in the last decade. Thirdly, this information was used to project the potential impact that scaling up essential interventions could have on reducing maternal and child mortality in South Africa. Finally, a costing exercise was undertaken to estimate the additional costs to the health system of scaling up these interventions.

The analysis used the LiST, whose modelling methods have been widely reviewed (20, 21). LiST is a module in a demographic software package called Spectrum, which compares the effect of different interventions on maternal, newborn and child deaths (22, 23). National baseline information on mortality rates and causes of death, background variables (e.g. fertility, economic status), current coverage of more than 60 interventions and their associated effectiveness values relative to specific causes of death and risk factors were used to estimate the deaths averted (24). The analysis was performed using Spectrum version 5.04.

**Fig. 1:**
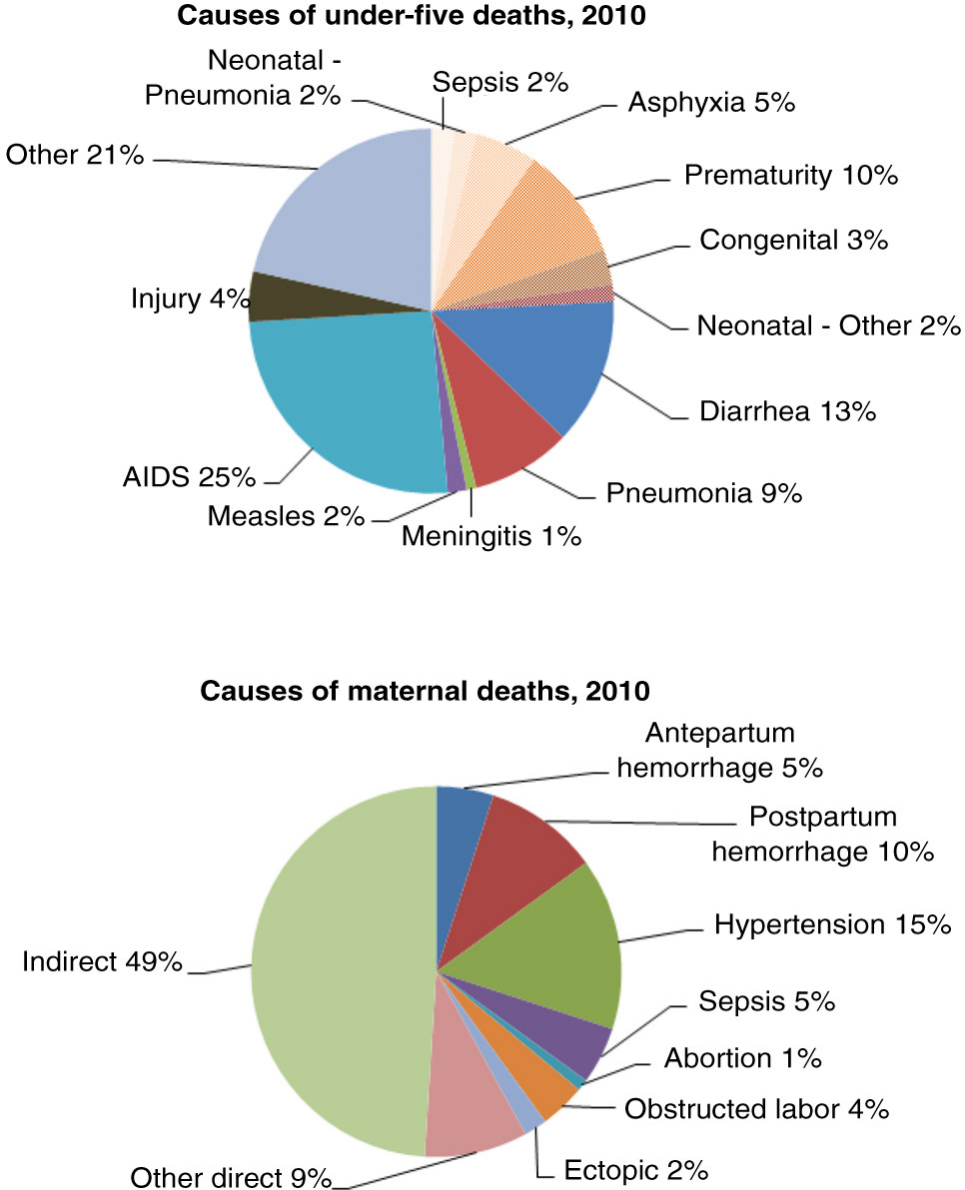
Causes of child, newborn and maternal deaths in SA used in the LiST model. Adapted from MRC BOD, 2010.

Taking 2010 as baseline year, we used an estimated national maternal mortality ratio (MMR) of 269 deaths per 100,000 live births (2), U5MR of 52.9/1,000 and neonatal mortality rate (NMR) of 13/1,000 (25). The causes of maternal, newborn and child mortality used were adapted from the South African Medial Research Council Burden of Disease unit estimates to fit the causal categories provided by LiST (9, 26), see Fig. 1.

### Analysing the model

The baseline coverage of interventions to reduce maternal, newborn and child mortality included in the model was reviewed and where possible compiled using national, population-based data (Table 1). Maternal, newborn and child deaths were then estimated, holding this baseline coverage constant, and compared to a scenario (which we call ‘achievable coverage’) where coverage of all interventions was scaled up to ambitious, yet possible levels in 2014 and 2015. Scale up of interventions in this achievable coverage scenario was based on consensus obtained from local experts across MCH on potentially attainable coverage levels in the next few years, taking into consideration recent policy changes, financial and resource inputs, and observed localised coverage change. The consultation with the experts took place at a 1-day meeting to discuss the trends in maternal and child interventions at the Wits School of Public Health in Johannesburg, South Africa. We invited 23 participants who work in the health sector in various positions at national and district level, clinical practice and academia. The participants were carefully selected among professionals who have been working in the MCH field for many years, and contribute to various programmes and specific interventions to improve maternal, newborn and child health in South Africa. In the absence of reliable and updated data on the coverage of health interventions in South Africa, we perceived the input of the experts to be critical, and reliable based on their experience. During the meeting, the participants were encouraged to deliberate on the coverage levels of each intervention, drawing on their own experiences. The discussions were facilitated by a member of the research team, following closely the methods employed in qualitative group assessments. The suggested coverage levels on each intervention were not adopted until consensus was reached.

**Table 1:** Baseline intervention coverage and achievable scale up by 2015, South Africa

Interventions	Baseline coverage (%)	Possible scale up (%)
Safe abortion services	35	45
Post abortion case management	60	80
Ectopic pregnancy case management	40	40
Antenatal care (4 visits)	50	65
TT – tetanus toxoid vaccination	77	77
Calcium supplementation	5	20
Hypertensive disease case management	40	50
Diabetes case management	10	10
MgSO_4_ – management of pre-eclampsia	75	85
Foetal growth restriction detection and management	10	15
Skilled birth attendance (SBA)	93	95
Facility delivery (clinic and hospital)	87	90
Unassisted deliveries	5	5
Basic emergency obstetric care (BEMOC)	5	10
Comprehensive emergency obstetric care (CEMOC)	50	75
All deliveries		
Clean birth practices	70	95
Immediate assessment and stimulation	70	72
Labour and delivery management	93	93
Neonatal resuscitation	40	50
Antenatal corticosteroids for preterm labour	20	40
Antibiotics for pPRoM	25	35
MgSO_4_ management of eclampsia	80	95
Active management of the third stage of labour	80	90
Induction of labour for pregnancies lasting 41+ weeks	10	10
Promotion of breastfeeding	25	35
Preventive postnatal care	10	20
Clean postnatal practices	10	10
Complementary feeding – education only	10	20
Complementary feeding – supplementation and education	5	15
Vitamin A supplementation	50	55
Improved water source	91	92
Water connection in the home	69	75
Improved sanitation – utilisation of latrines or toilets	74	80
Hand washing with soap	17	40
Hygienic disposal of children's stools	41	50
BCG	74	95
Polio	74	95
DPT	66	95
Hib	66	95
HepB	74	95
Pneumococcal	64	95
Rotavirus	66	95
Measles	74	95
Maternal Sepsis case management	75	85
Kangaroo mother care	25	40
Case management of severe neonatal infection	44	55
Injectable antibiotics	70	70
Full supportive care	44	60
ORS – oral rehydration solution	50	60
Antibiotics – for treatment of dysentery	80	80
Zinc – for treatment of diarrhoea	10	10
Oral antibiotics: case management of pneumonia in children	73	75
Vitamin A – for treatment of measles	75	75
Therapeutic feeding – for severe wasting	45	55
Treatment for moderate acute malnutrition	10	20

SBA=skilled birth attendance; BEMOC=basic emergency obstetric care; CEMOC=comprehensive emergency obstetric care.

The rate of scale up varied by intervention, and for certain interventions, experts indicated that ambitious coverage increases would not be possible in the short term. For example, labour and delivery management was maintained at 93% because this level was already high and unlikely to increase much more. Coverage of safe abortion services increased marginally, from 35 to 40% of those who need it, and breastfeeding promotion from 25 to 35%. Given current infrastructure and political will, coverage of all routine childhood vaccinations could increase to 95%.

Interventions that were not in the default version of LiST were created and added to the model, including treatment of childhood injuries (27), treatment of childhood TB (28), early detection and treatment of HIV in pregnant women (29) and inter-facility transport (30), Table 2. The interventions were assigned to specific causes of death that they could potentially affect. For example, early detection and treatment of HIV in pregnant women was associated with AIDS, an indirect maternal cause of death. Inter-facility transport was associated with direct maternal causes of death (e.g. ante- and post-partum haemorrhage). Intervention effectiveness was obtained from individual studies found in the literature (27–30).

**Table 2:** Interventions added to LiST

Intervention	Description	Baseline coverage (2010) (%)	Cause of death affected by the intervention	Effect estimate (%)	Affected fraction (%)
Treatment of childhood injuries (27)	Management of all paediatric trauma	50	Injury	50	100
Treatment of childhood TB (28)	Detection and treatment of childhood TB according to WHO guidelines (Rapid advice – 2010)	50	Pneumonia (TB is not split out as a separate cause of death in children in LiST)	99	2.5
Early detection and treatment of HIV in pregnant women	HIV treatment initiated before 20 weeks gestation	20	Indirect maternal	90	80
Treatment of pregnant women with TB (29)	Treatment of both latent infection and TB disease	50	Indirect maternal	90	50
Inter-facility transport for complicated deliveries (30)	Dedicated maternal ambulances for referral of complicated pregnancies between facilities	10	Direct maternal	30	80

LiST draws from several models within Spectrum, which provide assumptions on fertility, births, population HIV and other demographics. These include the demographic projections model (DemProj), family planning model (FamPlan) and the AIDS impact model (AIM). Changes were made to AIM, to reflect the current and projected coverage of HIV interventions. Spectrum defaults for family planning were retained.

### Costing methods

We modelled intervention costs using the costing module available in the Spectrum software. The module uses an ingredients approach to costing, based on four components: personnel and labour; drugs and supplies; other recurrent costs; and, capital costs. Staff remuneration is based on current salary structures of health workers in South Africa (31). The unit costs of drugs and supplies are based on international drug prices from UNICEF and the Management for Sciences Health International Drug Price Indicator (32, 33). The recurrent and capital portion of hospitalisation (in-patient days) is obtained from global estimates cost per patient day found in the WHO CHOICE database (24). Unit costs of outpatient visits are also obtained from the WHO-CHOICE database. Recurrent costs related to hospitalisation and outpatient visits include initial personnel training, gasoline, building rent, office supplies and promotional activities and publications (17, 34). Examples of indirect costs include maintenance workers, supervision of staff, insurance, utilities, and monitoring and evaluation. Costs estimated in LiST exclude infrastructure development, such as building clinics (17). The unit costs found in Spectrum were comparable to South African prices of drugs and supplies requested for tender by the Department of Health (35). All costs are presented in 2012 US dollars and per capita costs refer to the 2012 South African total population.

The costs provided in this paper were only for interventions for which information on unit costs is available in LiST. For example, the interventions we added to LiST (treatment of TB and injuries in children, inter-facility transport, treatment of TB and HIV in pregnancy) were not costed; other interventions not costed include water connection in the home, improved sanitation, case management of severe neonatal infection, micronutrient supplementation and treatment for moderate acute malnutrition.

### Estimating intervention cost-effectiveness

Cost-effectiveness was obtained by combining the health system cost of interventions and the lives saved impact calculated by LiST. We multiplied these lives saved by life expectancy to obtain the potential life years gained. The life expectancy at birth of 60 years was used for children and newborns. For mothers, we used the Reproductive-Aged Life Expectancy – RALE (36), which was estimated to be 27 years based on South Africa's 2011 life tables (37). Incremental intervention costs were then divided by incremental effects to obtain incremental cost-effectiveness ratios (ICERs), which show the additional costs required to achieve an additional unit of effect when interventions are scaled up. We adopted the WHO criteria on cost-effectiveness, which stipulate that an intervention is highly cost-effective if it averts a year of life lost for less than the national gross domestic product (GDP) per capita, cost-effective if one to three times the GDP per capita, and not cost-effective if greater than three times the GDP per capita (38). Given South Africa's GDP per capita (2012) of US$7,500 (39), interventions were considered highly cost-effective if the ICER was less than US$7,500, cost-effective if between US$7,500 and US$22,500, and not cost-effective if more than US$22,500.

## Results

### Reduction in child, newborn and maternal mortality by 2015

With current intervention coverage, U5MR was estimated to be 43 deaths/1,000 live births in 2013. The model projects that by 2015, U5MR could reduce by 21% to 34/1,000 and NMR by 31% to 9/1,000. MMR could be 154/100,000 (a reduction of about 43% from the 2013 estimate). These estimates, however, may still not be enough to meet the MDG targets.

### Child, newborn and maternal deaths averted

**Fig. 2:**
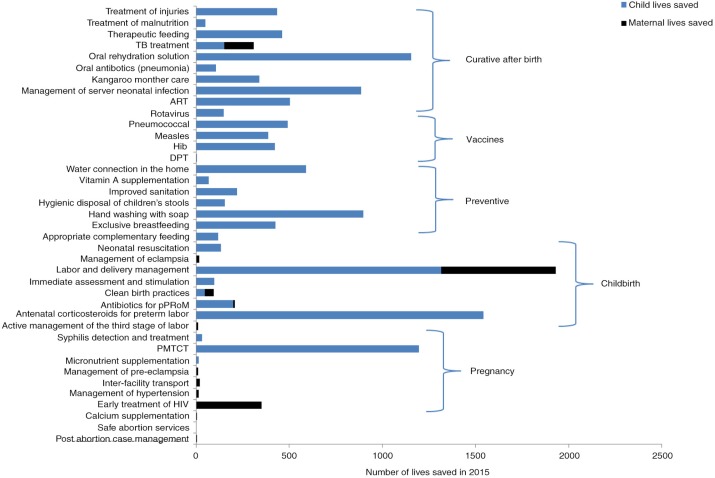
Number of lives saved in 2015 at achievable coverage.

If all interventions are scaled up to achievable coverage, an additional 12,500 newborn and child lives can be saved in 2015. Childbirth and curative after birth interventions will save the most lives, 3,300 and 2,900, respectively (Fig. 2). Antenatal corticosteroids for preterm labour (1,500) and labour and delivery management (1,300) account for the most lives saved. Oral rehydration solution (ORS) (1,100) and case management of severe neonatal infection (880) will save the most lives.

The top 11 interventions will account for 75% (9,400) of additional newborn and child lives saved (Table 3). Three interventions will account for about 30% of additional child lives saved: care for threatened preterm labour including antenatal corticosteroids, labour and delivery management and prevention of mother-to-child transmission (PMTCT) of HIV.

**Table 3:** Top 15 newborn, child and maternal interventions

	Newborn and child interventions			Maternal interventions		
	
	Intervention	Lives saved	% lives saved	Intervention	Lives saved	% lives saved
1	Antenatal corticosteroids for preterm labour	1,542	12	Labour and delivery management^a^	615	49
2	Labour and delivery management^a^	1,315	10	Early detection and treatment of HIV (pregnant women)	350	28
3	PMTCT	1,195	9	TB management in pregnant women	158	13
4	ORS – oral rehydration solution	1,153	9	Clean birth practices	47	4
5	Hand washing with soap	898	7	Inter-facility transport	20	2
6	Case management of severe neonatal infection	885	7			
7	Water connection in the home	591	5			
8	ART	503	4			
9	Pneumococcal	490	4			
10	Therapeutic feeding – for severe wasting	462	4			
11	Treatment of injuries	435				
	Total	9,469	75	Total	1,190	94

a Labour and delivery management counts towards saving both maternal and newborn lives.

Approximately 1,200 additional maternal lives will be saved by 2015. About 94% (1,100) of additional maternal lives saved will be accounted for by five interventions (Table 3). In total, 15 (labour and delivery management is counted twice) newborn, child and maternal interventions are expected to save an additional 10,600 lives per year (9,400 newborn and child; 1,100 maternal).

### Costs and cost-effectiveness

Using 2013 as the reference point, the baseline cost (2013 costs based on current coverage for all interventions included in the model, see Table 1), was estimated to be US$2.4 billion (US$44 per capita). This includes US$460 million for maternal and US$2.2 billion for newborn and child interventions. If interventions are scaled up to reach the levels in the achievable coverage scenario, an additional US$370 million (US$7 per capita) per year would be required (Table 5).

The additional costs of scaling up maternal health interventions are projected to be US$4.3 million (US$0.08 per capita) in 2015, with annual life years gained of 19,900 (Table 4). Pregnancy interventions will require annual incremental costs of about US$8 million. Hypertension management will have the smallest ICER (close to US$600). Clean birth practices (US$3.5 million) and active management of the third stage of labour (US$3.6 million) will require the most additional resources. The costs of labour and delivery management will reduce by over US$13 million per year, possibly due to a reduction in the number of pregnancies and births projected in future (40). Only one intervention, calcium supplementation, might not be cost-effective (ICER above US$22,500).

**Table 4:** Projected incremental costs and effects of maternal interventions in 2015

	Additional lives saved	Incremental costs (US$)	Life years gained	Cost (US$)/LY gained
Pregnancy				
Calcium supplementation	5	3,608,980	136	26,622
Hypertensive disease case management	13	204,209	352	579^a^
MgSO_4_ – management of pre-eclampsia	12	4,094,663	325	12,585^b^
Sub-total	30	7,907,851	813	
Childbirth				
Clean birth practices	47	3,439,886	1,274	2,699^a^
Labour and delivery management	615	−13,413,196	16,674	−804^a^
Antibiotics for pPRoM	10	361,066	271	1,332^a^
MgSO_4_ management of eclampsia	16	3,642,612	434	8,397^b^
Active management of the third stage of labour	10	2,413,870	271	8,903^b^
Sub-total	698	−3,555,762	18,924	
Total	728	4,352,089	19,737	

US$ = United States Dollars.

a Interventions highly cost-effective (less than US$7,500)

b interventions cost-effective (between US$7,500 and US$22,500). The following interventions are not included because unit cost estimates were not available: inter-facility transport, TB management and early detection and treatment of HIV in pregnancy, hence the total additional lives saved presented in this table are approximately half of the estimated 1,263 with achievable coverage.

The annual additional costs of interventions for newborns and children are expected to be over US$360 million (US$6 per capita), with over 600,000 life years gained per year (Table 5). The incremental costs will be US$280 million for preventive, US$33 million for vaccines, and US$50 million for HIV interventions. Most of the additional preventive costs will be accounted for by breastfeeding promotion, and the costs are largely driven by personnel costs required to ramp up breastfeeding prevalence from the current low levels. All newborn and child interventions are cost-effective.

**Table 5:** Projected incremental costs and effects of newborn and child interventions in 2015

Interventions	Additional lives saved	Incremental costs (US$)	Life years gained	Cost (US$)/LY gained
Childbirth				
Clean birth practices	46	3,439,886	2,760	1,246^a^
Immediate assessment and stimulation	98	4,145,317	5,880	705^a^
Labour and delivery management	1,315	−13,413,196	78,900	−170^a^
Neonatal resuscitation	134	20,853	8,040	3^a^
Antenatal corticosteroids for preterm labour	1,542	3,113,747	92,520	34^a^
Antibiotics for preterm rapture of membranes	198	361,066	11,880	30^a^
Sub-total	3,333	−2,332,327	199,980	
Preventive				
Breastfeeding promotion	427	227,904,210	25,620	8,896^b^
Appropriate complementary feeding	117	47,036,977	7,020	6,700^a^
Hand washing with soap	898	4,098,071	53,880	76^a^
Hygienic disposal of children's stools	154	1,802,872	9,240	195^a^
Sub-total	1,596	280,842,130	95,760	
Vaccines				
DPT	5	3,038,430	300	10,128^b^
Hib	423	3,506,228	25,380	138^a^
Pneumococcal	490	11,795,798	29,400	401^a^
Rotavirus	149	10,886,954	8,940	1,218^a^
Measles	386	4,488,290	23,160	194^a^
Sub-total	1,453	33,715,700	87,180	
Curative after birth				
Kangaroo mother care	338	523,723	20,280	26^a^
Oral rehydration solution	1,153	−9,609,885	69,180	−139^a^
Oral antibiotics: case management of pneumonia in children	107	−356,569	6,420	−56^a^
Therapeutic feeding – for severe wasting	462	14,709,388	27,720	531^a^
Sub-total	2,060	5,266,657	123,600	
HIV				
ART	503	61,368,745	30,180	2,033^a^
PMTCT	1,195	−11,038,195	71,700	−154^a^
Subtotal	1,698	50,330,550	101,880	
Total	10,140	367,822,710	608,400	

US$=United States Dollars.

a Interventions highly cost-effective (less than US$7,500)

b interventions cost-effective (between US$7,500 and US$22,500). The following interventions are not included because unit cost estimates were not available: TB and injury management, water connection in the home, improved sanitation, management of severe neonatal infection, micronutrient supplementation and treatment for moderate acute malnutrition, hence the total additional lives saved presented in this table are approximately half of the estimated 10,140 with achievable coverage.

The incremental costs of ORS, oral antibiotics and PMTCT are negative, implying that total costs of these interventions reduce over time. This is the result of increasing effective coverage of preventive interventions that affect the cause of death associated with curative interventions and reducing incidence of disease. For example, increasing the coverage of rotavirus vaccine and handwashing will reduce the number of diarrhoea infections, thereby reducing the number of children needing ORS, and associated costs of treatment.

### Scenario analysis

With 95% coverage, South Africa can be closer to MDG 4, but still far from attaining MDG 5. The model projects an NMR of 9/1,000, U5MR of 23/1,000 and MMR of 116/100,000 live births (Table 6). The additional lives saved will be 27,213 for newborns and children and 1,680 for mothers. The additional costs of scaling up interventions to 95% coverage will be US$2.1 billion per year.

**Table 6:** Selected outcomes of the scenario analysis with 95% coverage

Outcome	Estimate
Under 5 mortality rate (U5MR)	23/1,000
Neonatal mortality rate (NMR)	9/1,000
Maternal mortality rate (MMR)	116/100,000
Additional newborn and child lives saved	27,213
Additional maternal lives saved	1,680
Additional costs of scaling up to 95%	US$2.1 billion

The top interventions also differ when coverage is set at 95%. With 95% coverage, breastfeeding will save the most lives (3,000), followed by handwashing with soap (2,000), therapeutic feeding for wasting (1,680), corticosteroids (1,670) and water connection in the home (1,660).

## Discussion

South Africa has made progress towards reducing maternal and child mortality, but it is unlikely that this is sufficient to reach MDGs 4 and 5. In this analysis, assuming conservative intervention scale up, we project that an ambitious but realistic scenario for MDG 4 in the next 2 years will be a U5MR of 34/1,000, with NMR reducing to 9/1,000. For MDG 5, South Africa can attain an MMR of 153/100,000. In the scenario analysis, we show that if 95% coverage of interventions were possible, South Africa can attain a NMR of 9/1,000, U5MR of 23/1,000 and MMR of 116/100,000 live births.

Our estimated ratio of NMR to U5MR of 26% is lower than that estimated by others, such as the United Nations Inter-agency Group for Child Mortality Estimation (UN IGME), which sets the ratio at 40%. This may be due to different assumptions made regarding scale up, as well as which interventions will most likely save children's lives. These differences are likely to be greater in settings such as South Africa, which have high HIV prevalence. Usually, NMR reduces at a slower rate than U5MR, but in our analysis, we show the opposite. This might be because most of the interventions assessed in this analysis are facility based and will mostly affect neonates, while U5MR will be reduced by population based interventions, since a large percentage of under-five deaths in South Africa are at home (41).

We estimate that scaling up 15 key interventions will save an additional 10,000 lives of mothers and children by 2015. Saving maternal and newborn lives will largely be facility-based. In contrast, because close to 50% of deaths of children over 1 month of age occur at home (41), community-based health promotion interventions, such as breastfeeding and handwashing, will contribute significantly to further reductions in child mortality. Understanding this dichotomy is important to meeting both short- and long-term goals to reduce maternal, newborn and child mortality. Targeted interventions at facility level, for emergency obstetric care for example, can save lives of both mothers and babies (14). These interventions over which the NDOH has direct control can be prioritised in its strategic plans. Though we show that water, sanitation and hygiene programmes will have a high impact on child mortality (saving over 1,000 child lives annually) improving these interventions are multi-sectoral and will require more effort and time and will likely be more costly. Our results also show that treatment of HIV and TB early in pregnancy can save over 400 lives annually. Interventions for early detection and treatment of HIV and TB in pregnancy should be prioritised.

The resources to achieve these scenarios require careful consideration. We estimate that an additional US$370 million (US$7 per capita) will be required annually to scale up interventions with achievable coverage. Approximately US$0.10 for maternal health interventions and US$6 per capita for newborn and child health interventions will be required annually. Given South Africa's GDP per capita of US$7,500, all newborn and child health interventions are cost-effective (ICERs fall below US$15,000). Among the highly cost-effective interventions are labour and delivery management, PMTCT, kangaroo mother care and handwashing with soap (ICERs fall below US$7,500). All interventions among the 15 identified as highest impact, and for which costing was undertaken, were highly cost-effective. Of all the interventions considered in this analysis, only calcium supplementation may not be cost-effective according to our set criteria (ICER fell above US$22,500). The modelled impact of this intervention was also low, possibly because coverage of other interventions to prevent and manage pre-eclampsia and hypertension, for which calcium supplementation is said to be effective were estimated to be high, and might have lowered the impact of this intervention. One of the recommendations of the National Committee for the Confidential Enquiries into Maternal Deaths (NCCEMD) is for all facilities delivering ANC to provide calcium supplementation to all pregnant women (9) and it is known that maternal calcium supplementation has an additional benefit in preventing stillbirths (42). Innovative solutions and further research are needed to assess the feasibility and effect of alternative methods to address household food insecurity and food fortification.

The costing analysis likely represents a minimal estimate of the required resource envelope. The costing did not include costs largely outside the health sector for the improvement of water and sanitation, or the interventions that we added to LiST (inter-facility transport, treatment of TB and injuries and early treatment of HIV in pregnancy) because these are not pre-populated in the software. Costs related to infrastructure development were also not included. Taking these into account will drive the required additional costs upwards. However, the costs provided represent a close approximation of the direct medical or ‘health sector’ costs for the identified interventions for planning and prioritisation purposes.

An inherent assumption in the model is that increasing intervention coverage will lead to a reduction in maternal and child mortality. However, this may not necessarily be the case, as there are many factors that need to be taken into account, including the social determinants of health such as culture, migration, individual lifestyles and environmental factors (43–45). Other bottlenecks within the health system may also prevent changes in intervention coverage from having concomitant impact on mortality. These include logistics, training and health worker availability and motivation. Although South Africa meets the minimum density ratios for medical practitioners and professional nurses proposed by the WHO, there are major shortages created by unequal distribution in the public sector and rural areas. Rural areas have 44% of the population but are served by only 12% of the country's doctors. This inequity in the distribution of health workers is also seen across provinces; for example, the Western Cape Province has nine doctors per 10,000 population, compared to the North West province, which has about two doctors per 10,000. There is also a need for the training and re-training of some cadre of health workers, to enable them to understand how to deliver comprehensive services (46). Addressing these issues is key to scaling up essential maternal, newborn and child interventions, but this may require significant additional resources which have not been taken into account. However, none of the 15 interventions proposed in this analysis represent a new programme. All are already being implemented as part of integrated packages at some level across the country, but to achieve MDG 4 and 5 will need significantly more attention.

It is necessary to consider that cost savings can be made with increased efficiency in programme implementation. For example, task shifting, to allow mid-level health workers to perform some functions of doctors and nurses, can have significant benefits, as has been shown in countries such as Malawi, Mozambique and Tanzania, which allow trained and supervised mid-wives and medical assistants to perform caesarean sections (47, 48). At community level, postnatal visits can help save lives as well as reduce costs. Further, we show that increasing preventive interventions reduces the curative costs, and this should provide impetus for improving coverage.

Stillbirths have not been included in this analysis though many of the pregnancy and childbirth interventions would also prevent these deaths during pregnancy and during labour. Including stillbirths in the total number of lives saved would have a greater impact on the overall burden and a decreased cost per life saved (14).

### What is needed going forward?

Numerous solutions have been proposed for MCH in South Africa. Insight on the current challenges and detailed recommendations for action are provided in reports by the three inter-ministerial committees on MCH: the NCCEMD (9), National Perinatal Mortality and Morbidity Committee (NaPeMMCo) (49) and Committee on Morbidity and Mortality in Children under-5 years – CoMMiC (50). All of the 15 top interventions identified in our analysis have been recommended by these committees (Panel 1).

Panel 1 **Recommendations from inter-ministerial committees on maternal, perinatal and child mortality in South Africa**

The NCCEMD made 10 key recommendations in its Saving Mothers Report, for actions on the three major causes of maternal mortality: non-pregnancy related infections, obstetric haemorrhage and hypertension. The committee summarised its recommendations in five key points called the 5H's: 1) reducing deaths due to HIV/AIDS, through community mobilisation and ensuring facilities are able to screen for and initiate early treatment of HIV; 2) reducing deaths due to haemorrhage by promoting preventive interventions and practising active management of the third stage of labour; 3) reducing deaths due to hypertension through provision of calcium supplementation, early detection, referral and timely delivery; 4) training of health workers involved in maternity care on Essential Steps in the Management of Obstetric Emergencies (ESMOE), particularly using emergency obstetric simulation training – EOST; 5) health system strengthening to ensure 24-h access to functioning emergency obstetric care – provision of dedicated inter-facility transport, development of maternity waiting homes and standardised referral criteria among others.

NaPeMMCo has identified eight high impact interventions for 2013–2015 which could have a significant effect on the three main causes of neonatal deaths. These strategies include neonatal resuscitation; immediate assessment and stimulation; exclusive breastfeeding; immediate thermal care; clean birthing areas; hand washing with soap; kangaroo mother care; and full facility care. Suggested actions include effective monitoring of labour, provision of corticosteroids to every woman, and strict adherence to basic hygiene in labour wards.

CoMMiC recommended improving clinical care, placing emphasis on interventions at community and primary health care levels. CoMMiC has made specific recommendations for neonatal care, including readmitting sick newborns back into the nursery instead of children's wards. CoMMiC has also recommended increased emphasis on interventions at community and primary health care levels including an effective system for postnatal care. Improvement of health information systems, in order to support the notification and registration of deaths, will improve both planning as well as recognition of the magnitude of the current situation.

While these recommendations have been in place for several years, they have not been adequately implemented (51, 52). This will require concerted efforts to motivate health workers (53) and foster ownership and accountability at district and facility level.

It is essential to address supply and demand bottlenecks that prevent routine HIV and TB testing and treatment in pregnancy before 20 weeks (54). Stock outs of anti-retroviral drugs and TB medication, and other drugs and supplies must be carefully monitored, and government initiatives to improve communication between supplier, depots and facilities must be scaled up (55). Lessons can be learnt from other settings, where innovative interventions have helped to reduce demand-side barriers to health service access (56, 57).

In this analysis, we did not explore the potential impact of family planning, though it has been shown that family planning can contribute significantly to saving the lives of mothers and children (58). Family planning can help South Africa reduce its high teenage pregnancy rate (11), and subsequently prevent mortality in this high-risk group (59). The impact of family planning should be considered in future analyses.

As South Africa works towards achieving MDGs 4 and 5, family planning programmes should be strengthened, for example, by offering family planning education and contraceptives through community and school health programmes alongside public awareness campaigns (60). Addressing illegal abortions will require effective monitoring and evaluation of designated termination of pregnancy services, data capturing of illegal abortions, as well as the development of a national education strategy on termination of pregnancy.

There is a need to strengthen existing programmes to maintain immunisation achievements and to accurately capture coverage levels to determine which districts should be prioritised based on low coverage levels and high child population (61, 62).

A multi-sectoral approach between the Department of Health and the Department of Water Affairs as well as the Department of Local Government and Traditional Affairs (responsible for overseeing the functioning of local municipal councils), that harnesses collaboration to effectively implement water and sanitation programmes would ensure that water connection in the home is scaled up.

Lessons can be learnt from successful interventions in South Africa, such as the inter-facility transport intervention in the Free State province, that has resulted in a significant reduction in maternal mortality (30). Some provinces have already committed to improving inter-facility transport.

The coverage estimates in this analysis are based on current trends, policy and programme changes. Population-based coverage data for key interventions across the maternal, newborn and child health continuum of care have not been systematically captured since 2003. There is urgent need to strengthen data collection, including conducting regular demographic and health surveys with full pregnancy history information. Improving the scope and quality of routine health and cost information is key to assessment of progress and monitoring service delivery (63).

Finally, while national information can provide an overall picture, for greater traction in setting local targets and implementation of key interventions the analysis performed in this paper should be undertaken at provincial and district levels (64).

## Conclusions

South Africa has made progress in reducing maternal and child mortality. The analysis identified 15 key interventions, which were adopted by the NDOH as priorities in the time remaining to the MDG deadline in 2015. The Minister of Health then launched a campaign in all provinces, to garner support and encourage provincial health departments to scale up efforts to improve coverage of these 15 interventions, which were already being implemented in South Africa at varying levels of quality and coverage.

By scaling up these 15 effective interventions, an additional 10,000 maternal, newborn and child lives can be saved annually. While significant progress can be achieved for newborn and child health before the MDG deadline, addressing maternal mortality will continue to be challenging and South Africa will likely not be able to attain its MDG 5 target in the short term.

In order to make the most of the time left before the 2015 goal, efforts must focus on high impact, quality services delivered equitably, in line with recommendations made by the three inter-ministerial committees on maternal, perinatal and child health. Understanding the additional resources required, will enable appropriate budget bids to the National Treasury. We estimate that the additional cost of scaling up key interventions will amount to less than 1% of the annual national health budget. These costs do not seem out of reach for South Africa, considering that it has a per capita health expenditure of about US$645. We are however not certain whether these additional resources are available from the South African government. The 15 key interventions provided in this analysis can serve as a guide for managers and health workers on where to focus their efforts. Fostering ownership at facility level and empowering districts to readily respond to their challenges will be crucial. Improving maternal, newborn and child health should be a consultative process between the Department of Health and other stakeholders, as this will generate wide scale buy-in to decision making and priority setting. This study demonstrates the potential that priority setting tools such as the LiST can have in directing such processes.
